# CD27^+^IgD^−^ B cells in the peripheral blood of colorectal cancer patients: on anti-tumor or tumor-protective mission?

**DOI:** 10.18632/oncoscience.78

**Published:** 2014-09-09

**Authors:** Bernd Jahrsdörfer, Stefanie Lindner, Magdalena Hagn, Hubert Schrezenmeier

**Affiliations:** Institute of Transfusion Medicine, Ulm University, Ulm, Germany; Institute for Clinical Transfusion Medicine and Immunogenetics Ulm, Red Cross Blood Service Baden-Württemberg – Hessen, Germany

In their recent study published in *Oncotarget* Shimabukuro-Vornhagen and colleagues present interesting data on tumor-associated B cell subsets in patients with colorectal cancer [1]. The authors noted a significantly higher frequency of CD27^+^IgD^−^ B cells in the peripheral blood of such patients as compared to healthy subjects. The results were interpreted as a specific B cell immune response against the tumor, resulting in the accumulation of terminally differentiated memory B cells or plasma cells. Since the phenotype of B cells may not be sufficient to safely predict their function, we would like to suggest an alternative explanation for the occurrence of CD27^+^IgD^−^ B cells in these patients.

In a recent study, we screened the tumor microenvironment of various tumors for a novel regulatory B cell subset characterized by unique expression of the serine protease granzyme B (GrB) and potent GrBdependent T cell-suppressive activity [2]. We found that several tumor entities including colorectal, mamma, cervical and ovarian carcinomas contain significant numbers of GrB-expressing regulatory B cells. Notably, further phenotypic characterization of this GrB^+^ regulatory B cell subset showed enhanced expression of CD27, CD38, IgM, CD1d, CD86 and CD147. In contrast, expression of IgD and CD24 was downmodulated or unaltered in this novel regulatory B cell subset. The phenotype of GrB+ regulatory B cells is therefore in part similar to that of terminally differentiated plasma cells, a finding also reported by several independent groups working on distinct regulatory B cell subsets such as IL- 10-secreting regulatory B cells [3, 4].

**Figure 1:**
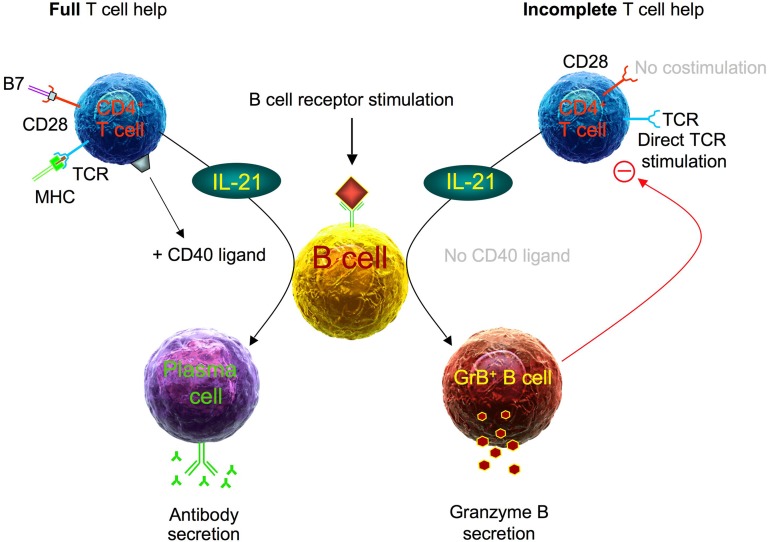
B cell differentiation in the presence of full T cell help as compared to incomplete T cell help Normal CD4^+^ T cell activation includes stimulation of both the TCR via MHC/peptide complexes and CD28 via B7 (left panel side). Such fully activated T cells secrete IL-21 and express high levels of CD40L, enabling them to induce plasma cell differentiation in B cells, which receive antigenspecific signals via their BCR at the same time. In contrast, early during viral infections and during malignant transformation the TCR of CD4^+^ T cells is often unspecifically stimulated via MHC-antigen complexes without simultaneous co-stimulation of CD28 (right panel side). Such incompletely activated T cells secrete IL-21, but barely express CD40L, resulting in the induction of GrB^+^ regulatory B cells.

The reason for this phenotypic similarity between regulatory B cells and plasma cells may be that both B cell populations share a key cytokine for their development, namely interleukin 21 (IL-21) [2, 5-7]. As previously shown by our group it depends on a second T cell-derived stimulus, CD40 ligand (CD40L), whether IL-21 drives B cells to differentiate into GrB-secreting regulatory B cells (in the absence of CD40L), or into antibody-secreting plasma cells (in the presence of CD40L) (Figure 1) [8, 9].

Meanwhile it is widely accepted that B cells exhibit a broad spectrum of functions beyond antibody secretion including T cell regulation, antigen presentation, cytokine production and direct cytotoxicity. Functional assays accompanying the phenotypic characterization of B cell populations may therefore avoid conflicting results on distinct functions of certain B cell subsets, particularly in an aberrant microenvironment such as in the presence of tumors.
